# A pilot randomized controlled trial comparing McKenzie and stabilization exercises for non‐specific neck pain in females aged 18–50 years: Effects on proprioception, pain, disability, and deep neck flexor endurance

**DOI:** 10.1002/ibra.70029

**Published:** 2026-09-07

**Authors:** Azzam Alarab, Abeer Alsharawi, Issam AbuQeis, Abeer Teeti, Osama Khodrog

**Affiliations:** ^1^ Department of Physiotherapy Palestine Ahliya University Bethlehem Palestine; ^2^ Institute of Neuroscience Kunming Medical University Kunming China; ^3^ Department of Radiology Palestinian Ministry of Health Ramallah Palestine; ^4^ Department of Epidemiology, School of Public Health Kunming Medical University Kunming China; ^5^ Department of Chemistry Hebron University Hebron Palestine; ^6^ Department of Medical Imaging and Diagnostic Radiology Palestine Technical University – Kadoorie Tulkarm Palestine

**Keywords:** cervical stabilization, McKenzie exercises, muscle endurance, non‐specific neck pain, proprioception

## Abstract

This pilot study compared the effects of McKenzie and cervical stabilization exercises on proprioception, pain, disability, and deep neck flexor endurance in females aged 18–50 years with non‐specific neck pain (NSNP). In this single‐blind, randomized controlled pilot trial, 40 females (18–50 years) with chronic NSNP were allocated to McKenzie (Group A, *n* = 20) or stabilization (Group B, *n* = 20). Outcomes were assessed pre/post‐intervention using JPE, VAS, NPADS, and deep neck flexor endurance tests. Both groups received three sessions/week for 4 weeks. Between‐group analyses used two‐way mixed ANOVA; ANCOVA was used to adjust for baseline differences in VAS and left‐rotation JPE. Effect sizes were reported as ηp^2^ and Cohen's *d*. Although both interventions produced clinically significant within‐group improvements across all measured outcomes, mixed ANOVA showed no significant Time × Group interactions for VAS, NPADS, endurance, or JPE flexion/extension/right rotation (all *p* > 0.05). A significant Time × Group interaction was initially observed for left‐rotation JPE (F(1,38) = 7.486, *p* = 0.009, *ηp* ^2^ = 0.165). However, after adjustment for the baseline imbalance, ANCOVA showed no significant between‐group difference in left‐rotation JPE, indicating comparable efficacy between interventions. ANCOVA also confirmed no significant between‐group difference in post‐intervention VAS after baseline adjustment. All participants (100%) achieved the Minimal Clinically Important Difference (MCID) (≥2‐point VAS reduction). In conclusion, both interventions are effective for NSNP, with comparable efficacy across most outcomes. After accounting for baseline differences, no significant between‐group superiority was demonstrated, including for left‐rotation proprioception.

## Graphical abstract

This pilot RCT compared McKenzie and stabilization exercises for non‐specific neck pain in young females. Both interventions significantly reduced pain and disability while improving proprioception and endurance, with comparable efficacy across most outcomes, though McKenzie showed superior left rotation proprioception.

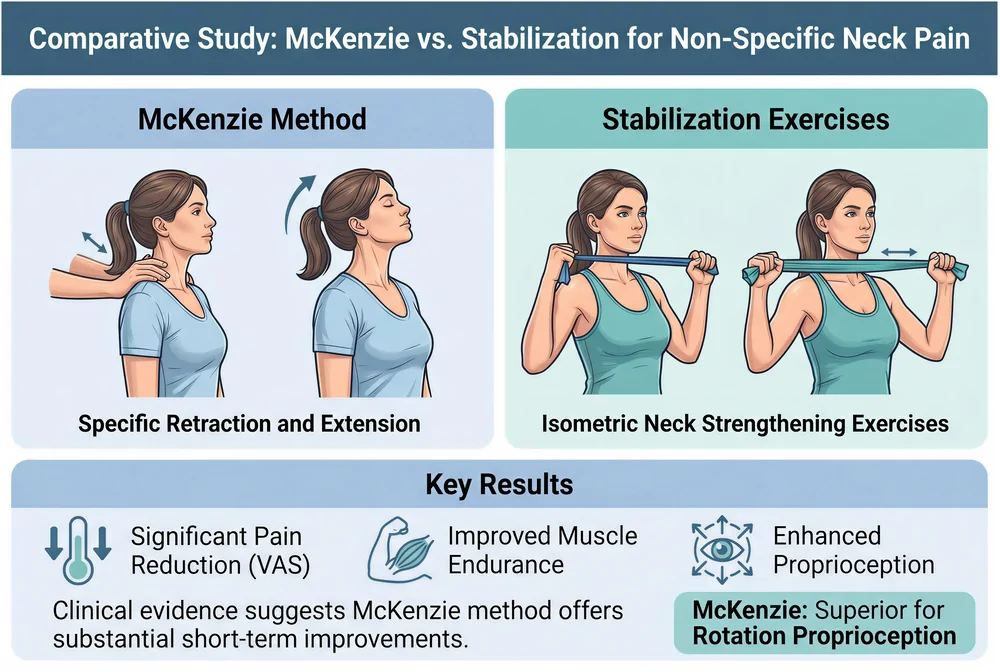

## 1 INTRODUCTION

Non‐specific neck pain (NSNP) is a prevalent musculoskeletal disorder that disproportionately affects women and frequently results in functional disability, work absenteeism, and diminished quality of life.^1^ The condition is biomechanically characterized by altered postural alignment, aberrant movement patterns, restricted cervical range of motion, and impaired proprioceptive awareness of the cervical musculature.^2^ Chronic NSNP, defined as symptoms persisting beyond 12 weeks, is often exacerbated by sedentary lifestyles, psychosocial stress, and poor ergonomics.^3^ Recent clinical investigations indicate that individuals with chronic neck discomfort frequently exhibit deficits in cervical proprioception, which compromises sensorimotor control. The cervical spine's proprioceptive system is integral to maintaining postural stability; it requires the continuous integration of visual, vestibular, and somatosensory inputs to coordinate the cervical musculature for optimal head alignment and joint stabilization.^4^ The clinical management of NSNP encompasses a variety of therapeutic modalities, including manual therapy, thermotherapy, and structured exercise regimens.^5^ Contemporary clinical guidelines strongly advocate for the inclusion of specific physical exercises as a primary intervention. These programs typically integrate cervical strengthening, range of motion activities, motor control training, and proprioceptive rehabilitation.^6^ Among these approaches, the McKenzie method (Mechanical Diagnosis and Therapy) is widely utilized for the assessment and management of mechanical neck pain. Conversely, cervical stabilization exercises have gained prominence for their focus on enhancing the neuromuscular coordination between the superficial and deep cervical muscles, thereby improving dynamic stability and reducing pain.^7^ Evidence suggests that targeted training of the deep cervical flexors can significantly ameliorate pain and enhance muscular endurance.^8^

Despite the established efficacy of exercise therapy, managing NSNP remains a significant clinical challenge, particularly in female populations who experience higher prevalence and chronicity.^9^ While numerous studies have investigated isolated therapeutic modalities, there is a paucity of research directly comparing the efficacy of the McKenzie method, a protocol primarily focused on mechanical realignment, with targeted cervical stabilization exercises designed to activate deep cervical musculature.^10^ Consequently, the comparative effectiveness of these interventions for improving deep neck muscle function and reducing pain in women with NSNP remains insufficiently understood. This pilot study addresses this gap by directly comparing the clinical outcomes of the McKenzie technique and cervical stabilization exercises, with a specific focus on their impact on deep cervical muscle function. The primary objective is to determine whether one approach yields superior improvements in proprioception, pain intensity, functional disability, and muscular endurance in females with chronic NSNP. By elucidating the specific benefits of these interventions, this research aims to inform the development of personalized, evidence‐based rehabilitation strategies that optimize functional recovery and mitigate the risk of symptom recurrence. This study was conducted at a single physiotherapy center where the available patient population during the recruitment period consisted predominantly of female referrals. Rather than artificially expanding inclusion criteria to capture male participants, we elected to focus on this homogeneous female cohort to maximize internal validity, acknowledging that this limits generalizability to male populations. This approach is consistent with the pilot study design, which prioritizes feasibility and effect size estimation over broad generalizability.

## 2 METHODS

### 2.1 Study design and participants

This single‐blind, randomized controlled pilot trial was conducted at the Al Abeer Physiotherapy Center in Dura‐Hebron from November 2023 to March 2024. The study protocol was approved by the Institutional Ethical Committee of the University of Ahliya (CAMS/PTBR/2/12/2024), and all procedures complied with the Declaration of Helsinki. Written informed consent was obtained from all participants before enrollment. The study enrolled 40 adult females, aged 18 to 50 years, who had been experiencing NSNP for a minimum of 12 weeks and were referred by an orthopedic physician. Exclusion criteria included individuals with specific neurological or systemic conditions, structural musculoskeletal abnormalities, or red‐flag symptoms such as unexplained weight loss or nocturnal pain. To ensure diagnostic accuracy and exclude cervical radiculopathy, participants underwent screening using the Spurling and Distraction tests. (detailed procedures provided in Supplementary File S1). Given the pilot nature of this study, a formal a priori sample size calculation was not performed. Instead, we targeted a minimum of 20 participants per group based on practical feasibility constraints and recommendations for pilot trials, which suggest 12–20 participants per group for preliminary effect size estimation.^11^ We acknowledge that this sample size is insufficient to detect small‐to‐medium between‐group differences, and the study is therefore exploratory rather than confirmatory.

### 2.2 Randomization, allocation concealment, and blinding

Following baseline assessments and confirmation of eligibility, participants were randomly allocated in a 1:1 ratio to either the McKenzie exercise group (Group A, *n* = 20) or the stabilization exercise group (Group B, *n* = 20). The randomization sequence was generated by an independent researcher (not involved in recruitment, treatment, or assessment) using computer software (SPSS v.31). Allocation concealment was maintained using sequentially numbered, opaque, sealed envelopes (SNOSE method). The enrolling physiotherapist opened the next envelope only after the participant had completed all baseline assessments and provided written informed consent. The randomization sequence remained concealed from all outcome assessors throughout the trial.

Given the nature of the exercise interventions, it was not possible to blind participants or treating physiotherapists to group allocation. However, the following measures were implemented to minimize bias: (1) Outcome assessments were conducted by a separate physiotherapist who was blinded to group assignment; (2) Participants were explicitly instructed not to discuss their intervention type with the assessor; (3) Treating physiotherapists were blinded to baseline outcome scores to prevent performance bias based on initial severity; (4) The statistician performing data analysis was blinded to group allocation until after the primary analysis was completed. We acknowledge that the absence of participant blinding may introduce performance and reporting bias, particularly for subjective outcomes (VAS, NPADS).

### 2.3 Interventions

Both groups completed three physiotherapy sessions per week for four consecutive weeks at the Al Abeer Physiotherapy Center.

#### 2.3.1 Group A (McKenzie exercises)

The protocol focused on mechanical diagnosis and therapy principles, primarily utilizing cervical retraction and extension movements to centralize symptoms and restore mobility.

#### 2.3.2 Group B (Stabilization exercises)

The intervention targeted the deep cervical flexors and extensors through multi‐directional isometric exercises and resistance training using Thera‐bands to enhance neuromuscular control and dynamic stability. Detailed descriptions of both exercise protocols, including specific repetitions and progression criteria, are provided in Supplementary File S2.

### 2.4 Outcome measures

Clinical outcomes were evaluated at baseline and immediately following the 4‐week intervention period. The primary outcome measures included:

#### 2.4.1 Visual analog scale (VAS)

A 10‐cm continuous scale used to assess current pain intensity, where 0 represents no pain, and 10 represents maximum imaginable pain.^12^

#### 2.4.2 Neck pain and disability scale (NPADS)

A comprehensive 20‐item questionnaire evaluating the impact of neck pain on functional activities, emotional state, and mobility.^13^

#### 2.4.3 Joint position error (JPE)

A measure of cervical proprioception assessed using a laser pointer method. Participants attempted to actively relocate their heads to a neutral starting position after maximal cervical movements, with eyes closed. Errors were recorded in degrees for flexion, extension, and bilateral rotation.^14^, ^15^

#### 2.4.4 Deep neck flexor endurance test

Evaluated by timing the participant's ability to maintain a supine, active chin‐tuck position with the head elevated approximately 2.5 cm above the examination table.^16^, ^17^ Detailed procedures for all outcome assessments are available in Supplementary File S1.

### 2.5 Statistical analysis

Statistical analyses were performed using IBM SPSS Statistics (v. 31). Baseline comparability was assessed using independent samples *t*‐tests and chi‐square tests. Normality of change scores was assessed using the Shapiro–Wilk test. Endurance changes scores deviated from normality in both groups (Group A: *p* = 0.001; Group B: *p* = 0.047). Given the balanced sample sizes (*n* = 20 per group) and the robustness of ANOVA to moderate deviations from normality, the mixed ANOVA was retained for the between‐group comparison of endurance. Primary between‐group analyses employed two‐way mixed ANOVA (time × group), with Greenhouse–Geisser corrections applied when sphericity was violated. Effect sizes were reported as partial eta‐squared (*ηp* ^2^). Analysis of Covariate Adjustment (ANCOVA) was conducted for post‐intervention VAS with baseline scores as covariates. Within‐group changes were analyzed using paired *t*‐tests with Cohen's *d* effect sizes (G*Power v3.1.9.4). The Minimal Clinically Important Difference (MCID) was defined as a ≥2‐point VAS reduction. A *p*‐value < 0.05 was considered significant.

## 3 RESULTS

### 3.1 Participant flow and adherence

**FIGURE 1:**
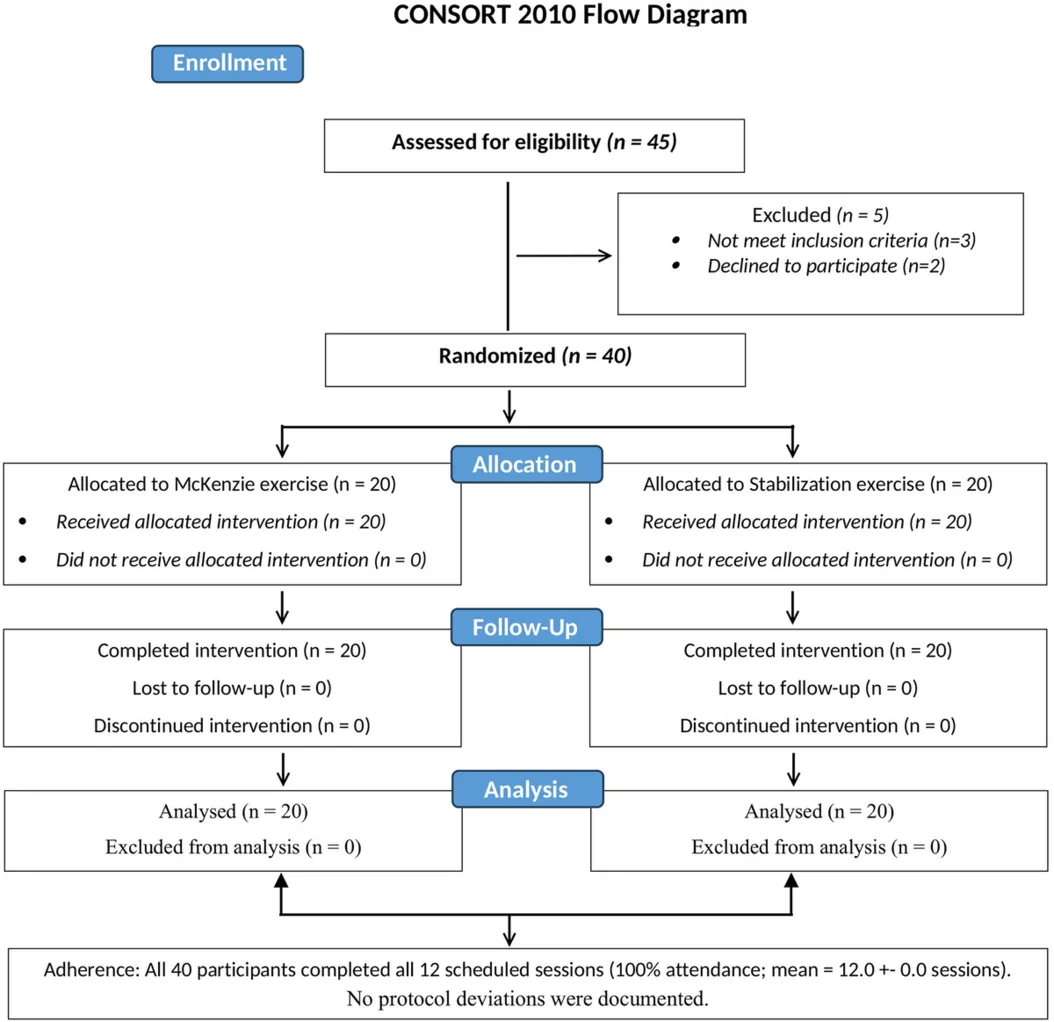
CONSORT 2010 flow diagram showing participant enrollment, allocation, follow‐up, and analysis. All 40 randomized participants completed the 4‐week intervention with 100% session attendance. No participants were lost to follow‐up or excluded from analysis. [Color figure can be viewed at wileyonlinelibrary.com]

A CONSORT 2010 flow diagram is presented in Figure 1. Of the 45 patients screened for eligibility, 5 were excluded (3 did not meet the inclusion criteria; 2 declined to participate). The remaining 40 participants were randomized (20 per group), received their allocated intervention, completed all 12 scheduled sessions, and were included in the final analysis. The attendance rate was 100% (mean = 12.0 ± 0.0 sessions), and no protocol deviations were documented. While this perfect retention is encouraging, it is atypical for chronic pain samples and may reflect the short intervention duration, frequent monitoring, and personalized attention in this single‐center pilot trial, limiting generalizability to less controlled settings.

### 3.2 Baseline characteristics

The study included 40 female participants, evenly divided between the McKenzie group (*n* = 20) and the stabilization group (*n* = 20). As shown in Table 1, the groups were well matched at baseline with respect to age distribution (*p* = 0.93) and employment status (*p* = 0.50), confirming successful randomization. Baseline clinical characteristics for all outcome measures are presented in Supplementary Table S3. While most baseline values were comparable between groups, a statistically significant difference was observed in baseline VAS scores (Group A: 7.80 ± 0.77 vs. Group B: 8.25 ± 0.64, *p* = 0.035) and baseline JPE left rotation (Group A: 4.80 ± 1.33° vs. Group B: 6.06 ± 1.63°, *p* = 0.042). These differences were addressed through covariate adjustment in subsequent analyses.

**TABLE 1:** Baseline demographic characteristics of the study participants.

	Group A (McKenzie, *n* = 20)			Group B (Stabilization, *n* = 20)			
Characteristic	Category	N	Percentage	Category	N	Percentage	*p* value
Age (years)	18–28:	11	(55%)	18–28:	9	(45%)	0.93
	29–39:	3	(15%)	29–39:	8	(40%)	
	40–50:	6	(30%)	40–50:	3	(15%)	
Employment	Employed:	9	(45%)	Employed:	10	(50%)	0.50
	Unemployed:	11	(55%)	Unemployed:	10	(50%)	

*Note*: Data are presented as number (%). No statistically significant differences were observed between the McKenzie and stabilization groups at baseline (*p* > 0.05), confirming successful randomization.

### 3.3 Primary between‐group analyses

**FIGURE 2:**
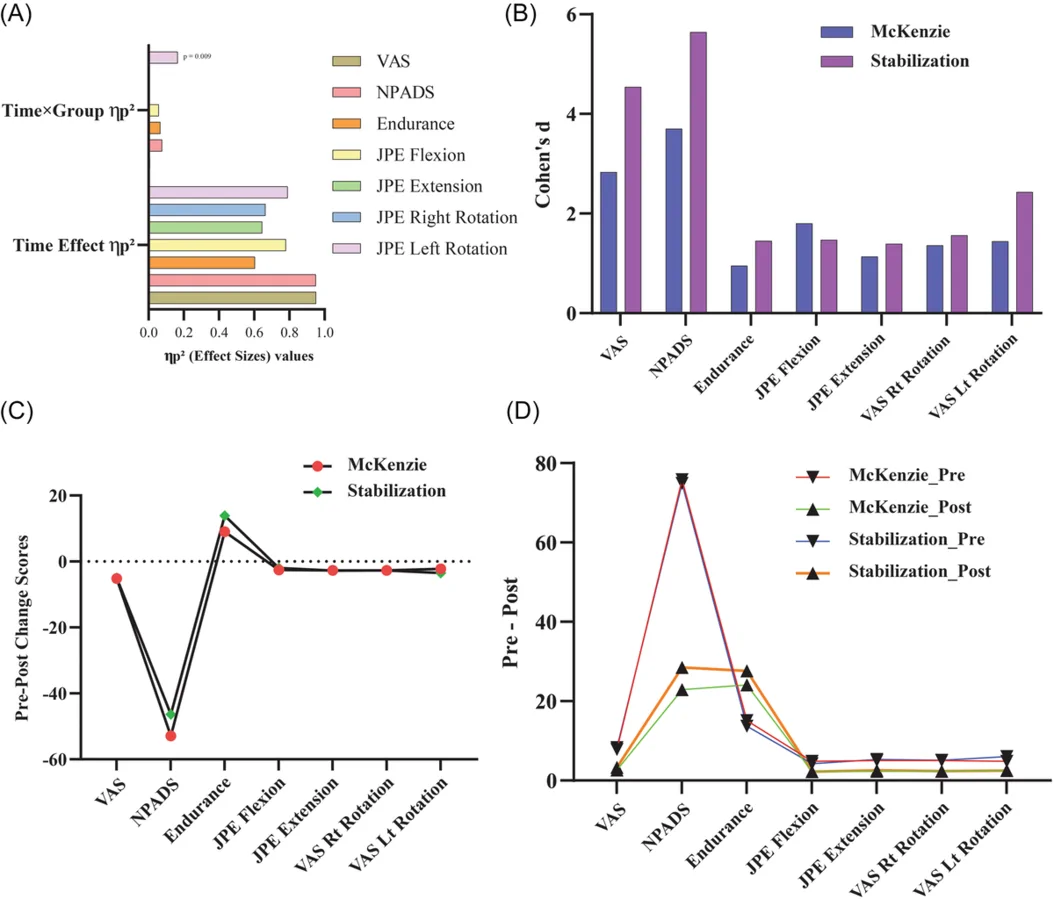
Between‐group and within‐group treatment effects of McKenzie and stabilization exercises for non‐specific neck pain. (A) Partial eta‐squared (*ηp* ^2^) values from a two‐way mixed ANOVA showing the Time Effect (all p < 0.001) and Time × Group Interaction. (B) Within‐group Cohen's d effect sizes for McKenzie (blue) and stabilization (purple) interventions across all outcomes. (C) Pre‐post mean change scores (Δ) for McKenzie (red circles) and stabilization (green diamonds), with the dotted line at zero indicating no change. (D) Raw pre‐ and post‐intervention scores for McKenzie (red/green lines) and stabilization (blue/orange lines), illustrating parallel improvement trajectories. DNF, Deep Neck Flexor; JPE, Joint Position Error; NPADS, Neck Pain and Disability Scale; VAS, Visual Analog Scale. [Color figure can be viewed at wileyonlinelibrary.com]

Two‐way mixed ANOVAs were conducted to evaluate the effects of time (pre/post), group (McKenzie vs. stabilization), and their interaction on all outcome measures. Figure 2A illustrates the between‐group comparison of effect sizes (*ηp* ^2^), showing comparable Time × Group interactions across six of seven outcomes, except for JPE left rotation, where McKenzie demonstrated superior improvement (*F* (1,38) = 7.486, *p* = 0.009, *ηp* ^2^ = 0.165). Complete ANOVA output for all outcomes is provided in Supplementary Table S4. All variables demonstrated significant main effects of time (*p* < 0.001), confirming substantial within‐group improvements across both interventions. The Time × Group interaction was non‐significant for VAS (F (1,38) = 0.295, *p* = 0.590, *ηp* ^2^ = 0.008), NPADS (F (1,38) = 3.156, *p* = 0.084, *ηp* ^2^ = 0.077), endurance (F (1,38) = 2.717, *p* = 0.108, *ηp* ^2^ = 0.067), flexion JPE (F (1,38) = 2.340, *p* = 0.134, *ηp* ^2^ = 0.058), extension JPE (F (1,38) = 0.004, *p* = 0.948, *ηp* ^2^ < 0.001), and right rotation JPE (F (1,38) = 0.001, *p* = 0.976, *ηp* ^2^ < 0.001), indicating comparable efficacy between groups for these outcomes. However, a significant Time × Group interaction was observed for left rotation JPE (F (1,38) = 7.486, *p* = 0.009, *ηp* ^2^ = 0.165), with the McKenzie group demonstrating superior improvement in left rotation proprioception compared to the stabilization group (Table 2).

**TABLE 2:** Two‐way mixed ANOVA results for between‐group comparisons.

Outcome measure	Time Effect F (1,38)	Time × Group Interaction F (1,38)	*p* value	*ηp* ^2^	Interpretation
VAS (0–10)	752.70, *p* < 0.001	0.295	0.590	0.008	No between‐group difference
NPADS (0–100)	724.54, *p* < 0.001	3.156	0.084	0.077	Trend (not significant)
DNF Endurance (s)	57.90, *p* < 0.001	2.717	0.108	0.067	No between‐group difference
JPE – Flexion (°)	135.73, *p* < 0.001	2.340	0.134	0.058	No between‐group difference
JPE – Extension (°)	69.23, *p* < 0.001	0.004	0.948	<0.001	No between‐group difference
JPE – Right Rotation (°)	75.24, *p* < 0.001	0.001	0.976	<0.001	No between‐group difference
**JPE – Left Rotation (°)**	**143.51, *p*** < **0.001**	**7.486**	**0.009**	**0.165**	**Significant: McKenzie superior**

*Note*: All time main effects were significant at *p* < 0.001. *ηp* ^2^ = partial eta‐squared (effect size). Bold and shaded row indicates statistically significant interaction.

Abbreviations: DNF, Deep Neck Flexor; JPE, Joint Position Error; NPADS, Neck Pain and Disability Scale; VAS, Visual Analog Scale.

### 3.4 Covariate‐adjusted analysis

Given the significant baseline difference in VAS scores between groups (Group A: 7.80 ± 0.77 vs. Group B: 8.25 ± 0.64, *p* = 0.035), an ANCOVA was conducted to compare post‐intervention VAS scores while controlling for baseline VAS. The baseline VAS score was a significant covariate (F(1,37) = 4.986, *p* = 0.032, *ηp* ^2^ = 0.119). After adjustment for baseline VAS, there was no statistically significant between‐group difference in post‐intervention pain (F(1,37) = 1.034, *p* = 0.316, *ηp* ^2^ = 0.027). The estimated marginal means were 2.78 (95% CI: 2.25–3.31) for the McKenzie group and 3.17 (95% CI: 2.64–3.70) for the stabilization group (Table 3). Similarly, given the significant baseline imbalance in JPE left rotation (Group A: 4.80 ± 1.33° vs. Group B: 6.06 ± 1.63°, *p* = 0.042), an ANCOVA was conducted comparing post‐intervention JPE left rotation scores between groups while controlling for baseline values. The covariate (baseline JPE left rotation) was not a significant predictor of post‐intervention outcomes (F(1,37) = 1.856, *p* = 0.181, *ηp* ^2^ = 0.048). After adjusting for baseline differences, there was no significant between‐group difference in post‐intervention left rotation proprioception (F(1,37) = 0.555, *p* = 0.461, *ηp* ^2^ = 0.015), with estimated marginal means of 4.18° (95% CI: 3.61–4.75) for McKenzie and 3.87° (95% CI: 3.30–4.44) for stabilization (Table 4).

**TABLE 3:** ANCOVA results for post‐intervention VAS, controlling for baseline.

Source	Type III SS	df	Mean square	*F*	*p* value	*ηp* ^2^
Corrected Model	10.727	2	5.363	4.113	0.024	0.182
Intercept	0.870	1	0.870	0.667	0.419	0.018
VAS_Pre (Covariate)	6.502	1	6.502	4.986	0.032	0.119
Group	1.349	1	1.349	1.034	0.316	0.027
Error	48.248	37	1.304			
Total	413.000	40				

*Note*: *R* ^2^ = 0.182 (Adjusted *R* ^2^ = 0.138). Dependent variable: VAS_Post.

Abbreviation: VAS, Visual Analog Scale.

**TABLE 4:** ANCOVA results for post‐intervention JPE_LtRotation, controlling for baseline.

Source	Type III SS	df	Mean Square	F	*p*‐value	ηp^2^
Corrected Model	35.660	3	11.887	0.952	0.395	0.049
Intercept	32.185	1	32.185	22.325	<0.001	0.376
JPE_LtRotation_Pre (Covariate)	2.675	1	2.675	1.856	0.181	0.048
Group	0.800	1	0.800	0.555	0.461	0.015
Error	53.342	37	1.442			
Total	89.002	40				

*Note*: *R* ^2^ = 0.049 (Adjusted *R* ^2^ = −0.002). Dependent variable: JPE_LtRotation_Post.

Abbreviation: JPE_LtRotation, Joint Position Error left rotation scores.

### 3.5 Within‐group treatment effects

Both intervention groups demonstrated statistically significant within‐group improvements across all measured outcomes following the 4‐week intervention (all *p* < 0.001). Figure 2B presents the within‐group Cohen's *d* effect sizes, revealing large to very large effects for both McKenzie (*d* = 0.95–3.70) and stabilization (*d* = 1.45–5.64) interventions. Figure 2C,D depicts the pre‐post change scores, illustrating the parallel trajectories of improvement between groups. Detailed within‐group pre‐ and post‐intervention data, including mean changes, 95% confidence intervals, and effect sizes, are presented in Supplementary Table S5. In the McKenzie group, mean VAS score decreased from 7.8 to 2.6 (*p* < 0.001, *d* = 2.83), and NPADS improved from 75.8 to 22.9 (*p* < 0.001, *d* = 3.70). Cervical proprioception (JPE) improved in all directions with large effect sizes (*d* = 1.14 to 1.80). Deep neck flexor endurance increased from 15.1 to 24.1 s (*p* < 0.001, *d* = 0.95). Similarly, the stabilization group exhibited significant reductions in VAS (8.3 to 3.3, *p* < 0.001, *d* = 4.54) and NPADS (74.9 to 28.5, *p* < 0.001, *d* = 5.64), alongside substantial gains in JPE across all planes (*p* < 0.001) and deep neck flexor endurance (13.7 to 27.6 s, *p *< 0.001, *d* = 1.45). Normality testing of change scores (Supplementary Table S6) revealed that endurance change violated normality assumptions (Group A: W = 0.811, *p* = 0.001; Group B: W = 0.903, *p* = 0.047), confirming the appropriate use of non‐parametric tests for this outcome. All other change scores were normally distributed (*p* > 0.05).

### 3.6 Clinical relevance

Clinical relevance was assessed using the MCID, defined as a ≥2‐point reduction on the VAS. All participants (100%) in both the McKenzie group and the stabilization group achieved this threshold. However, in the absence of a control group, this high response rate cannot be definitively attributed to the interventions rather than natural history, placebo effects, or regression to the mean. The MCID achievement rate should therefore be interpreted as descriptive rather than evidence of treatment‐specific efficacy. Statistical comparison of success rates was not feasible (Table 5).

**TABLE 5:** Minimal clinically important difference (MCID) achievement.

Group	Achieved MCID (*n*)	Did Not Achieve (*n*)	Achievement rate
Group A (McKenzie)	20	0	100.0%
Group B (Stabilization)	20	0	100.0%
Total	40	0	100.0%

*Note*: MCID is defined as ≥2‐point reduction on VAS. Chi‐square test not computable due to constant distribution (no variation in outcomes).

## 4 DISCUSSION

This randomized controlled pilot trial compared the efficacy of McKenzie exercises and cervical stabilization exercises on proprioception, pain intensity, functional disability, and deep neck flexor endurance in females aged 18–50 years with chronic NSNP. The principal finding is that both interventions produced statistically and clinically significant within‐group improvements across all measured outcomes (all *p* < 0.001), with large‐to‐very‐large effect sizes. Importantly, between‐group analyses revealed comparable efficacy for six of seven outcomes, with one exception: the McKenzie group demonstrated superior improvement in left rotation proprioception compared to the stabilization group. These findings provide preliminary evidence supporting exercise‐based management of NSNP in a female‐specific cohort. Both exercise protocols yielded substantial within‐group improvements in pain intensity and functional disability. In the McKenzie group, VAS decreased by 5.2 points (from 7.8 to 2.6, *d* = 2.83) and NPADS improved by 52.9 points (from 75.8 to 22.9, *d* = 3.70).

The stabilization group showed similarly large improvements, with VAS decreasing by 5.0 points (from 8.3 to 3.3, *d* = 4.54) and NPADS improving by 46.4 points (from 74.9 to 28.5, *d* = 5.64). The two‐way mixed ANOVA confirmed non‐significant Time × Group interactions for both VAS (*F*(1,38) = 0.295, *p* = 0.590, *ηp* ^2^ = 0.008) and NPADS (*F*(1,38) = 3.156, *p* = 0.084, ηp^2^ = 0.077), indicating comparable efficacy. Given the significant baseline difference in VAS scores (Group A: 7.80 ± 0.77 vs. Group B: 8.25 ± 0.64, *p* = 0.035), we conducted an ANCOVA with baseline VAS as a covariate. After adjustment, the between‐group difference remained non‐significant (*F*(1,37) = 1.034, *p* = 0.316), with estimated marginal means of 2.78 (95% CI: 2.25–3.31) for McKenzie and 3.17 (95% CI: 2.64–3.70) for stabilization. The 100% MCID achievement rate, while encouraging, must be interpreted with substantial caution. In the absence of a control or placebo group, we cannot distinguish intervention effects from natural history, placebo responses, or regression to the mean. Chronic non‐specific neck pain is known to exhibit fluctuating symptoms, and spontaneous improvement rates of 30–50% over 4‐week periods have been reported in observational studies.^18^ Furthermore, the threshold for MCID achievement (≥2‐point VAS reduction) was modest given the high baseline pain scores (7.8–8.3/10), making it statistically likely that even minor improvement would cross this threshold. The absence of variation (no participant failed to achieve MCID) precluded statistical comparison of success rates. Future definitive trials should include a control group to establish treatment‐specific effects on MCID achievement.

These findings align with Mahmoud Abadi et al.,^19^ who demonstrated that neck stabilization and thoracic mobility exercises significantly reduce disability and improve endurance in females with chronic NSNP. Similarly, Aman et al.^20^ emphasized the effectiveness of proprioceptive training for improving motor function and sensorimotor control. Our results extend this evidence by directly comparing two distinct exercise philosophies, mechanical diagnosis versus neuromuscular stabilization, and demonstrating equivalent clinical outcomes, a novel contribution given the paucity of head‐to‐head trials in this domain. Cervical proprioception, assessed using Joint JPE, improved significantly in both groups across all movement planes (all *p* < 0.001). Although the initial mixed ANOVA showed a significant Time × Group interaction for left rotation JPE (F(1,38) = 7.486, *p* = 0.009, *ηp* ^2^ = 0.165), this effect was no longer significant after adjustment for the baseline imbalance using ANCOVA (F(1,37) = 0.555, *p* = 0.461, ηp^2^ = 0.015). The initial between‐group signal may therefore have been influenced by the significant baseline difference in left rotation JPE (4.80° vs. 6.06°, *p* = 0.042). Thus, the present findings do not provide sufficient evidence for a direction‐specific superiority of the McKenzie method in left rotation proprioception. Both groups achieved significant gains in deep neck flexor endurance (both *p* < 0.001). The stabilization group exhibited a numerical advantage, increasing from 13.7 to 27.6 s (Δ = +13.9 s, *d* = 1.45) compared to the McKenzie group's increase from 15.1 to 24.1 s (Δ = +9.0 s, *d* = 0.95). However, the Time × Group interaction did not reach statistical significance (*F*(1,38) = 2.717, *p* = 0.108, *ηp* ^2^ = 0.067), indicating that this difference cannot be confidently attributed to intervention type rather than sampling variability. Notably, normality testing revealed that endurance change scores violated distributional assumptions in both groups (Shapiro–Wilk: Group A *p* = 0.001; Group B *p* = 0.047), necessitating non‐parametric testing for this outcome and reducing statistical power to detect subtle between‐group differences.

The minimal detectable change (MDC) for cervical JPE using the laser pointer method has been reported as approximately 2.5°–4.2° depending on movement direction and testing condition, with a standard error of measurement (SEM) ranging from 0.8° to 2.3°. Given that the observed between‐group difference in JPE left rotation change scores was approximately 1.3° (McKenzie: −2.21° vs. stabilization: −3.53°), this falls within the range of measurement error for this outcome. Consequently, even statistically significant differences may not represent clinically meaningful proprioceptive improvements. The trend toward greater endurance gains in the stabilization group is biomechanically plausible, as this protocol explicitly targets the deep cervical flexors through multi‐directional isometric exercises and Thera‐band resistance training. Nevertheless, the significant endurance improvement in the McKenzie group (*d* = 0.95) indicates that mechanical therapy, despite its primary focus on symptom centralization and range restoration, indirectly enhances muscular endurance, likely by optimizing cervical posture and reducing aberrant loading patterns that contribute to premature fatigue. This is consistent with Gumuscu et al.,^21^ who reported that various exercise training improves outcomes in chronic neck pain, and with Abdel‐Aziem et al.,^22^ who found that both McKenzie and deep neck flexor exercises are effective for chronic neck pain management. This study offers several novel contributions. First, it is among the few randomized trials to directly compare McKenzie and stabilization exercises in a homogeneous female cohort with chronic NSNP, addressing a population known to experience higher prevalence and chronicity of neck pain. Second, the study highlights the importance of accounting for baseline differences when interpreting between‐group proprioceptive outcomes. Although the initial analysis suggested a direction‐specific difference in left rotation JPE, this apparent effect was no longer significant after adjustment for the baseline imbalance using ANCOVA, indicating that the initial between‐group signal may have been influenced by baseline differences. Third, the demonstration that both approaches achieve 100% MCID success rates provides compelling evidence for their clinical utility in conservative management.

Our findings partially contrast with those of Gumuscu et al.,^21^ who reported differential outcomes across three exercise protocols in chronic neck pain. However, their study did not include a direct McKenzie versus stabilization comparison, limiting comparability. Similarly, Abdel‐Aziem et al.^22^ found that McKenzie protocol and deep neck flexor exercises compelling evidence outcomes, but their design did not isolate the stabilization component as distinctly as our Thera‐band resistance protocol. The comparable efficacy observed in our trial is consistent with structured, evidence‐based exercise, regardless of specific theoretical orientation, and constitutes a cornerstone of NSNP management. Recent evidence suggests potential synergistic benefits of combined approaches. Amin et al.^23^ demonstrated that a hybrid program incorporating both McKenzie and stabilization exercises was superior to McKenzie alone for pain reduction and range of motion. While our study did not test a combined intervention, the comparable efficacy of the individual protocols suggests that sequential or integrated programming may capitalize on the distinct mechanistic strengths of each approach: mechanical restoration from McKenzie and neuromuscular endurance from stabilization. Future research should explore optimal sequencing or dosing of such combined protocols. The significant endurance gains observed in both groups are noteworthy, given the established role of deep neck flexor dysfunction in chronic neck pain. Demir et al.^24^ reported that therapeutic and stabilization exercises after manual therapy improve outcomes in non‐specific chronic neck pain, supporting our finding that active exercise is essential for functional recovery. Furthermore, Chaiyawijit and Kanlayanaphotporn^25^ found that McKenzie neck exercises improved deep neck flexor strength and endurance comparably to cranio‐cervical flexion exercises, which aligns with our observation that mechanical therapy indirectly enhances muscular endurance despite not explicitly targeting these muscles. Both proprioception and muscular endurance improved within each group, consistent with previous exercise intervention studies.

## 5 LIMITATIONS

Several limitations must be acknowledged. First, the sample was restricted to adult females aged 18–50 years, limiting generalizability to males, older adults, and occupational subgroups. Second, the absence of a true control or placebo group precludes definitive attribution of improvements solely to the interventions, as natural history and placebo effects cannot be entirely excluded. Third, the lack of long‐term follow‐up prevents assessment of durability. Fourth, the pilot study design with *n* = 20 per group may have been underpowered to detect subtle between‐group differences, particularly for endurance, where a trend was observed (*p* = 0.108). Fifth, the JPE left rotation finding is that the observed between‐group difference in change scores (approximately 1.3°) falls within the reported standard error of measurement (SEM ≈ 0.8°–2.3°) and minimal detectable change (MDC ≈ 2.5°–4.2°) for cervical proprioception assessment using the laser pointer method. Therefore, the statistical significance of this interaction may not translate to a clinically meaningful difference in sensorimotor function. Future studies should employ more precise proprioception assessment tools (e.g., inertial measurement units) to reduce measurement error.

Finally, the significant baseline differences in VAS and left rotation JPE, though addressed statistically via ANCOVA, introduce some uncertainty regarding group equivalence at enrollment. The inability to blind participants and treating physiotherapists to group allocation is a significant limitation. Although the outcome assessor was blinded, the lack of participant blinding may have inflated subjective outcomes (VAS, NPADS) through expectancy effects. Future definitive trials should consider sham or attention‐control exercise conditions to enable participant blinding. Additionally, within‐group effect sizes observed in this trial (Cohen's d up to 5.64) are notably larger than those typically reported in exercise intervention studies for chronic neck pain (typically *d* = 0.5–1.5). Several factors may contribute to this inflation: (1) the absence of a control group precludes estimation of non‐specific effects (placebo, natural history, regression to the mean); (2) the lack of participant blinding to treatment allocation may have enhanced expectancy effects for subjective outcomes (VAS, NPADS); (3) the high baseline scores may have amplified the apparent magnitude of change. These effect sizes should be interpreted as descriptive of the observed within‐group changes rather than as unbiased estimates of treatment efficacy.

## 6 CONCLUSION

This pilot study provides preliminary evidence that both McKenzie exercises and cervical stabilization exercises produce within‐group improvements in pain, disability, proprioception, and deep neck flexor endurance in females aged 18–50 years with chronic NSNP. Between‐group comparisons suggest comparable efficacy across most outcomes, though the apparent superiority of McKenzie for left rotation proprioception requires confirmation in a fully powered trial with appropriate baseline adjustment. The absence of a control group and the small sample size preclude definitive conclusions about treatment‐specific efficacy. These findings support the feasibility of a larger, definitive randomized controlled trial and suggest that exercise‐based interventions warrant continued investigation for NSNP management.

## AUTHOR CONTRIBUTIONS

Azzam Alarab contributed to conceptualization, methodology, project administration, and supervision. Abeer Alsharawi handled the investigation, data curation, and prepared the initial draft. Issam AbuQeis and Abeer Teeti provided software, performed statistical analysis, and handled validation, visualization, and manuscript revision. Osama Khodrog contributed to data curation, supervision, and writing, review & editing. All authors read and approved the final manuscript.

## CONFLICT OF INTEREST STATEMENT

The authors declare no conflicts of interest.

## ETHICS STATEMENT

The Institutional Ethical Committee of the University of Ahliya gave its approval to the study procedure (CAMS/PTBR/2/12/2024). The number for the Universal Trial (UTN) is U1111‐1317‐3601. All of the procedures were carried out in compliance with the Declaration of Helsinki, and patients gave their informed consent before any collection.

## Supporting information

Supplementary File S1.

Supplementary File S2.

Supplementary Table S3_1.

Supplementary Table S4_1.

Supplementary Table S5_1.

Supplementary Table S6_1.

## Data Availability

The data that support the findings of this study are available from the corresponding author upon reasonable request.
