# Supplementary material for: A pilot randomized controlled trial comparing McKenzie and stabilization exercises for non‐specific neck pain in females aged 18–50 years: Effects on proprioception, pain, disability, and deep neck flexor endurance
Source: Ibrain. 2026 Sep 7;12(3):290–301. doi: 10.1002/ibra.70029 (PMC13548818; doi:10.1002/ibra.70029)
Supplement: Supplementary file 1 [file IBRA-12-290-s001.docx]

**Supplementary Table S3.** Baseline clinical characteristics

| **Outcome Measure** | **Group A (McKenzie)** | **Group B (Stabilization)** | **p-value** |
| --- | --- | --- | --- |
| VAS (0-10) | 7.80 ± 0.77 | 8.25 ± 0.64 | 0.035* |
| NPADS (0-100) | 75.80 ± 11.92 | 74.85 ± 7.84 | 0.376 |
| DNF Endurance (s) | 15.10 ± 5.11 | 13.70 ± 6.94 | 0.569 |
| JPE Flexion (°) | 4.82 ± 1.75 | 4.21 ± 1.14 | 0.424 |
| JPE Extension (°) | 5.00 ± 2.22 | 5.33 ± 1.68 | 0.391 |
| JPE Rt Rotation (°) | 5.00 ± 1.97 | 5.08 ± 1.52 | 0.804 |
| JPE Lt Rotation (°) | 4.80 ± 1.33 | 6.06 ± 1.63 | 0.042* |

***Note:*** *Baseline clinical characteristics of participants in the McKenzie exercise group (Group A, n = 20) and the cervical stabilization exercise group (Group B, n = 20). Values are presented as mean ± standard deviation. Between-group comparisons were performed using independent samples t-tests.*
