# Supplementary material for: A pilot randomized controlled trial comparing McKenzie and stabilization exercises for non‐specific neck pain in females aged 18–50 years: Effects on proprioception, pain, disability, and deep neck flexor endurance
Source: Ibrain. 2026 Sep 7;12(3):290–301. doi: 10.1002/ibra.70029 (PMC13548818; doi:10.1002/ibra.70029)
Supplement: Supplementary file 2 [file IBRA-12-290-s002.docx]

**Supplementary Table S6. Normality testing (Shapiro-Wilk)**

| **Outcome Measure** | **Group A Statistic** | **p-value** | **Group B Statistic** | **p-value** |
| --- | --- | --- | --- | --- |
| Endurance\_Change | 0.811 | **0.001** | 0.903 | **0.047** |
| NPADS\_Change | 0.933 | 0.176 | 0.943 | 0.277 |
| VAS\_Change | 0.915 | 0.078 | 0.929 | 0.147 |
| JPE\_LtRotation\_Change | 0.973 | 0.826 | 0.944 | 0.285 |
| JPE\_RtRotation\_Change | 0.932 | 0.169 | 0.955 | 0.457 |
| JPE\_Extension\_Change | 0.914 | 0.077 | 0.915 | 0.081 |
| JPE\_Flexion\_Change | 0.952 | 0.404 | 0.975 | 0.846 |

**Note:** Shapiro-Wilk normality tests for change scores (post-intervention minus pre-intervention) in the McKenzie exercise group (Group A, n = 20) and the cervical stabilization exercise group (Group B, n = 20).
