# Supplementary material for: A pilot randomized controlled trial comparing McKenzie and stabilization exercises for non‐specific neck pain in females aged 18–50 years: Effects on proprioception, pain, disability, and deep neck flexor endurance
Source: Ibrain. 2026 Sep 7;12(3):290–301. doi: 10.1002/ibra.70029 (PMC13548818; doi:10.1002/ibra.70029)
Supplement: Supplementary file 3 [file IBRA-12-290-s003.docx]

**Supplementary File S1. Detailed Clinical Assessment Procedures**

**S1.1 Diagnostic Screening Tests**

To ensure the accurate diagnosis of non-specific neck pain (NSNP) and exclude cervical radiculopathy, the following provocative tests were utilized during the initial screening:

1. Spurling Test: The participant is seated comfortably. The examiner passively rotates and laterally flexes the participant's head toward the affected side. A downward compressive force is then applied from the apex of the head. A positive test is indicated by the reproduction of radiating discomfort or radicular symptoms extending into the ipsilateral arm. This test demonstrates high specificity (94%) and sensitivity (95%) for cervical radiculopathy.

2. Distraction Test: The examiner stands behind the seated participant and places one hand on the participant's forehead and the other on the occiput (alternatively, the hands can be cupped over the mastoid processes). An upward distracting force is applied while slightly flexing the neck. A positive test is recorded if the participant reports an alleviation or reduction of radicular pain during the traction. This test has a specificity of 90% and a sensitivity of 44%.

**S1.2 Outcome Measurement Protocols**

1. Joint Position Error (JPE) Test: This test evaluates cervical proprioception. A lightweight laser pointer is securely affixed to the participant's head. The participant is seated 90 centimeters away from a target on a wall. The initial resting position of the laser projection is marked as the "target center." The participant is instructed to close their eyes (or wear a blindfold), actively move their head to a maximal range in a specific direction (flexion, extension, right rotation, or left rotation), and then attempt to accurately return their head to the initial starting position. The distance between the final laser point and the target center is measured to calculate the error angle. Three repetitions are performed for each direction, and the average error is recorded for analysis.

2. Deep Neck Flexor Endurance Assessment: The participant is positioned supine in a hook-lying posture. The examiner instructs the participant to perform an active chin tuck (craniocervical flexion) and then lift their head and neck approximately 2.5 cm (1 inch) off the examination table while maintaining the chin tuck. The examiner places a hand on the table directly beneath the participant's occiput. The endurance time is recorded in seconds. The test is terminated if the participant's occiput rests on the examiner's hand for more than one second, or if the skin folds on the anterior neck begin to separate, indicating a loss of the chin tuck.
