# Supplementary material for: A pilot randomized controlled trial comparing McKenzie and stabilization exercises for non‐specific neck pain in females aged 18–50 years: Effects on proprioception, pain, disability, and deep neck flexor endurance
Source: Ibrain. 2026 Sep 7;12(3):290–301. doi: 10.1002/ibra.70029 (PMC13548818; doi:10.1002/ibra.70029)
Supplement: Supplementary file 4 [file IBRA-12-290-s004.docx]

**Supplementary Table S5.** Within-group pre-post comparisons

| **Outcome** | **Group A (McKenzie)** |  |  |  | **Group B (Stabilization)** |  |  |  |
| --- | --- | --- | --- | --- | --- | --- | --- | --- |
|  | **Pre → Post** | **Δ** | **p** | **d** | **Pre → Post** | **Δ** | **p** | **d** |
| VAS | 7.8→2.6 | 5.2 | <0.001 | 2.83 | 8.3→3.3 | 5 | <0.001 | 4.54 |
| NPADS | 75.8→22.9 | 52.9 | <0.001 | 3.7 | 74.9→28.5 | 46.4 | <0.001 | 5.64 |
| Endurance | 15.1→24.1 | 9 | <0.001 | 0.95 | 13.7→27.6 | 13.9 | <0.001 | 1.45 |
| JPE Flexion | 4.82→2.23 | 2.59 | <0.001 | 1.8 | 4.21→2.23 | 1.98 | <0.001 | 1.47 |
| JPE Extension | 5.00→2.28 | 2.72 | <0.001 | 1.14 | 5.33→2.57 | 2.76 | <0.001 | 1.39 |
| JPE Rt Rotation | 5.00→2.28 | 2.72 | <0.001 | 1.36 | 5.08→2.37 | 2.71 | <0.001 | 1.56 |
| JPE Lt Rotation | 4.80→2.59 | 2.21 | <0.001 | 1.44 | 6.06→2.53 | 3.53 | <0.001 | 2.43 |

***Note:*** *Within-group pre- and post-intervention comparisons for the McKenzie exercise group (Group A, n = 20) and the cervical stabilization exercise group (Group B, n = 20), including mean change scores (Δ), p-values, and Cohen's d effect sizes.*
