# Supplementary material for: A pilot randomized controlled trial comparing McKenzie and stabilization exercises for non‐specific neck pain in females aged 18–50 years: Effects on proprioception, pain, disability, and deep neck flexor endurance
Source: Ibrain. 2026 Sep 7;12(3):290–301. doi: 10.1002/ibra.70029 (PMC13548818; doi:10.1002/ibra.70029)
Supplement: Supplementary file 5 [file IBRA-12-290-s005.docx]

**Supplementary File S2. Detailed Exercise Intervention Protocols**

Both intervention groups completed a 5-minute warm-up and a 5-minute cool-down consisting of general neck and upper extremity stretching during each session. The primary exercise protocols lasted approximately 30 minutes.

**S2.1 Group A: McKenzie Exercise Protocol**

Participants performed the following sequence of exercises, focusing on mechanical realignment:

Head Retraction (Seated): The participant retracts the head horizontally backward without tilting the chin up or down. Hold for 10 seconds. Repeat 10 times.

Supine Neck Extension: The participant lies supine with the head extending off the edge of the bed, allowing gravity to gently extend the neck. Repeat 10 times.

Seated Neck Extension: Following retraction, the participant extends the neck backward as far as comfortable. Repeat 10 times.

Neck Rotation (Seated): The participant actively rotates the head to the left and right limits of motion. Repeat 10 times per side.

Lateral Bending (Seated): The participant actively bends the neck, bringing the ear toward the shoulder on both the left and right sides. Repeat 10 times per side.

Neck Flexion (Seated): The participant actively flexes the neck, bringing the chin toward the chest. Repeat 10 times [19].

**S2.2 Group B: Stabilization Exercise Protocol**

Participants performed a combination of deep neck flexor activation and isometric resistance exercises using a Thera-band [17, 18]:

Deep Neck Flexor Activation (Supine): The participant performs a chin tuck and holds the position for 5 seconds. Repeat 10 times.

Multi-directional Isometrics (Seated): The participant applies manual resistance against their own head in flexion, extension, lateral bending, and rotation without allowing movement. Hold for 5 seconds. Repeat 3 times for each direction.

Thera-band Cervical Extension Isometric: The band is wrapped around the back of the head. The participant holds the ends in front and pushes the elbows forward to create tension while maintaining a neutral neck posture with a slight chin tuck. Hold for 5 seconds. Repeat 5 times.

Thera-band Cervical Side-bending Isometric: The band is wrapped around the side of the head. The participant holds the ends on the opposite side to create tension while maintaining a neutral neck posture. Hold for 5 seconds. Repeat 5 times per side.

Thera-band Cervical Flexion Isometric: The band is wrapped across the forehead. The participant holds the ends behind the head to create tension while maintaining a neutral neck posture. Hold for 5 seconds. Repeat 5 times.

Thera-band Cervical Rotation Isometric: The band is wrapped around the back of the head, and the ends are crossed over the forehead. The participant pulls the band laterally to create rotational resistance while maintaining a stable neck. Hold for 5 seconds. Repeat 5 times per side.
