# Supplementary material for: A pilot randomized controlled trial comparing McKenzie and stabilization exercises for non‐specific neck pain in females aged 18–50 years: Effects on proprioception, pain, disability, and deep neck flexor endurance
Source: Ibrain. 2026 Sep 7;12(3):290–301. doi: 10.1002/ibra.70029 (PMC13548818; doi:10.1002/ibra.70029)
Supplement: Supplementary file 6 [file IBRA-12-290-s006.docx]

**Supplementary Table S4.** Complete ANOVA output

| **Source** | **Outcome** | **Type III SS** | **df** | **Mean Sq** | **F** | **p-value** | **ηp²** |
| --- | --- | --- | --- | --- | --- | --- | --- |
| Time | VAS | 510.05 | 1 | 510.05 | 752.695 | <0.001 | 0.952 |
| Time × Group | VAS | 0.2 | 1 | 0.2 | 0.295 | 0.59 | 0.008 |
| Time | NPADS | 49252.81 | 1 | 49252.81 | 724.537 | <0.001 | 0.95 |
| Time × Group | NPADS | 214.512 | 1 | 214.512 | 3.156 | 0.084 | 0.077 |
| Time | Endurance | 2610.612 | 1 | 2610.612 | 57.899 | <0.001 | 0.604 |
| Time × Group | Endurance | 122.512 | 1 | 122.512 | 2.717 | 0.108 | 0.067 |
| Time | JPE Flexion | 104.425 | 1 | 104.425 | 135.73 | <0.001 | 0.781 |
| Time × Group | JPE Flexion | 1.8 | 1 | 1.8 | 2.34 | 0.134 | 0.058 |
| Time | JPE Extension | 150.23 | 1 | 150.23 | 69.234 | <0.001 | 0.646 |
| Time × Group | JPE Extension | 0.009 | 1 | 0.009 | 0.004 | 0.948 | <0.001 |
| Time | JPE Rt Rotation | 146.975 | 1 | 146.975 | 75.238 | <0.001 | 0.664 |
| Time × Group | JPE Rt Rotation | 0.002 | 1 | 0.002 | 0.001 | 0.976 | <0.001 |
| Time | JPE Lt Rotation | 165.055 | 1 | 165.055 | 143.512 | <0.001 | 0.791 |
| Time × Group | JPE Lt Rotation | 8.609 | 1 | 8.609 | **7.486** | **0.009** | **0.165** |

***Note:*** *Two-way mixed analysis of variance (ANOVA) results examining the main effect of time (pre- vs. post-intervention) and the Time × Group interaction for all outcome measures. Degrees of freedom for all F-tests: numerator = 1, denominator = 38.*
